# Ulinastatin ameliorated streptozotocin-induced diabetic nephropathy: Potential effects via modulating the components of gut-kidney axis and restoring mitochondrial homeostasis

**DOI:** 10.1007/s00424-023-02844-6

**Published:** 2023-08-10

**Authors:** Fatma H. Rizk, Amira A. El Saadany, Marwa Mohamed Atef, Rania Nagi Abd-Ellatif, Dina M. El-Guindy, Muhammad T. Abdel Ghafar, Marwa M. Shalaby, Yasser Mostafa Hafez, Shaimaa Samir Amin Mashal, Eman H. Basha, Heba Faheem, Ramez Abd-Elmoneim Barhoma

**Affiliations:** 1 Department of Physiology, Faculty of Medicine, Tanta University, Tanta, Egypt; 2 Department of Pharmacology, Faculty of Medicine, Tanta University, Tanta, Egypt; 3 Department of Medical Biochemistry, Faculty of Medicine, Tanta University, Tanta, Egypt; 4 Department of Pathology, Faculty of Medicine, Tanta University, Tanta, Egypt; 5 Department of Clinical Pathology, Faculty of Medicine, Tanta University, Tanta, Egypt; 6 Department of Medical Microbiology and Immunology, Faculty of Medicine, Tanta University, Tanta, Egypt; 7 Department of Internal Medicine, Faculty of Medicine, Tanta University, Tanta, Egypt

**Keywords:** Ulinastatin, Diabetic nephropathy, Gut-kidney axis, Mitochondrial homeostasis

## Abstract

Growing evidence supports the role of the gut-kidney axis and persistent mitochondrial dysfunction in the pathogenesis of diabetic nephropathy (DN). Ulinastatin (UTI) has a potent anti-inflammatory effect, protecting the kidney and the gut barrier in sepsis, but its effect on DN has yet to be investigated. This study aimed to assess the potential mitigating effect of UTI on DN and investigate the possible involvement of gut-kidney axis and mitochondrial homeostasis in this effect. Forty male Wistar rats were divided equally into four groups: normal; UTI-treated control; untreated DN; and UTI-treated DN. At the end of the experiment, UTI ameliorated DN by modulating the gut-kidney axis as it improved serum and urinary creatinine, urine volume, creatinine clearance, blood urea nitrogen, urinary albumin, intestinal morphology including villus height, crypt depth, and number of goblet cells, with upregulating the expression of intestinal tight-junction protein claudin-1, and counteracting kidney changes as indicated by significantly decreasing glomerular tuft area and periglomerular and peritubular collagen deposition. In addition, it significantly reduced intestinal and renal nuclear factor kappa B (NF-κB), serum Complement 5a (C5a), renal monocyte chemoattractant protein-1 (MCP-1), renal intercellular adhesion molecule 1 (ICAM1), and renal signal transducer and activator of transcription 3 (STAT3), mitochondrial dynamin related protein 1 (Drp1), mitochondrial fission 1 protein (FIS1), mitochondrial reactive oxygen species (ROS), renal hydrogen peroxide (H_2_O_2_), and 8-hydroxy-2'-deoxyguanosine (8-OHdG) levels. Furthermore, it significantly increased serum short chain fatty acids (SCFAs), and mitochondrial ATP levels and mitochondrial transmembrane potential. Moreover, there were significant correlations between measured markers of gut components of the gut-kidney axis and renal function tests in UTI-treated DN group. In conclusion, UTI has a promising therapeutic effect on DN by modulating the gut-kidney axis and improving renal mitochondrial dynamics and redox equilibrium.

## Introduction

Diabetes mellitus (DM) is the main cause of end-stage renal disease worldwide, with diabetic nephropathy (DN) affecting 40% of diabetic patients [4]. DN increases the risk of comorbidities such as cardiovascular disease [39]. Evidence suggests that chronic low-grade inflammation plays a role in the pathogenesis of DN as chemokines, proinflammatory cytokines, and cell adhesion molecules were found to be highly expressed in diabetic patients' renal tissues [35].

Growing evidence suggests that the gut-kidney axis plays a pivotal role in the pathogenesis of DN [27]. In induced diabetic model of rats, previous study reported that there were increases in the metabolites of abnormal gut flora, activation of renin-angiotensin (RAS) system, and impairment of renal function. Nonetheless, these effects could be reversed when using broad-spectrum antibiotics, indicating that DM can lead to renal injury by activating the RAS system with the metabolites of abnormal intestinal flora in diabetic rats [27]. In addition, DM leads to structural and functional abnormalities in the intestinal mucosal barrier allowing the intestinal bacteria to pass through the leaky intestinal barrier and stimulate an immune response [9, 21]. Bacteria will be phagocytized by the accumulating macrophages, which will then release inflammatory factors. The inflammatory factors and bacterial metabolites then enter the bloodstream and go to the kidneys, where they induce oxidative stress and inflammation, thereby damaging endothelial cells and podocytes (4). Therefore, the gut-kidney axis could be a potential target for DN management [42].

Moreover, oxidative stress and disrupted redox equilibrium play critical roles in the progression of chronic inflammation in DN. They are caused mainly by persistent mitochondrial dysfunction. Therefore, it is thought that pharmacologically targeting the inflammatory process and regulating mitochondrial networking are promising therapeutic strategies for preventing and restoring normal kidney function in DN [45].

Ulinastatin, a urinary trypsin inhibitor (UTI), is a glycoprotein derived from human urine. Due to its inhibitory effect on cytokines and other proinflammatory mediators, UTI was used in many clinical trials to treat patients with acute pancreatitis [17] and protecting kidneys in patients with septic shock [41] and ischemia [40]. Furthermore, because of its anti-inflammatory properties, UTI may have had beneficial effects on the gut barrier in the early stages of sepsis [20] as well as on isoflurane-induced cell apoptosis and mitochondrial damage in vitro [24]. However, to the best of our knowledge, the effects of UTI on DN, the gut-kidney axis, and renal mitochondrial dynamics have not been investigated. Therefore, this study aimed to assess the potential mitigating effect of UTI on DN and the possible role of the gut-kidney axis and mitochondrial homeostasis in this effect.

## Materials and methods

### Drugs and chemicals

Streptozotocin (STZ) was obtained from Sigma-Aldrich (St. Louis, MO, USA) and was freshly prepared and dissolved in 0.1 M cold citrate buffer (pH: 4.5). UTI solution was purchased from Bharat serums and vaccines co., Ltd. (Ambernath, India) and dissolved in normal saline. All other chemicals used in this study were purchased from Sigma-Aldrich Co. (St. Louis, MO, USA) and were of high analytical grade.

### Animals

Male Wistar rats weighing 180–200 g were obtained from the official animal supplier of the Faculty of Science, Tanta University, and housed in cages under controlled conditions (25˚C and a 12 h light/dark cycle), with ad libitum access to standard rodent chow (EL-Nasr Chemical Company, Cairo, Egypt) and water. The rats were acclimatized for two weeks before the start of the experiment.

### Induction of DN

After acclimatization, in all rats except the normal and UTI-treated groups, DM was induced by intraperitoneal (i.p) injection with a single dose of STZ (60 mg/kg) to overnight-fasted rats. The initial STZ-induced hypoglycaemia in the first 24 h was overcome by giving STZ-treated rats 5% glucose solution instead of water. Three days after STZ injection, fasting blood glucose (FBG) levels were measured in a tail vein sample using a glucometer (LifeScan, CA, USA, catalog no. 022896). Rats with high FBG (> 250 mg/dl) were used for subsequent experiments. After four weeks, 24-h urine samples were collected from all diabetic rats using metabolic cages, centrifuged at 3000 × g for 5 min to remove impurities, and stored at − 80 °C until analysis. Successful DN induction, based on the results of a previous study, was considered in rats with hyperglycemia (≥ 250 mg/dl) and proteinuria (≥ 8.0 mg/dl) who were selected for further study [2]. Progressive albuminuria, decrease in renal function and the characteristic histological changes in the glomeruli and the tubulointerstitial lesions are observed in human DN. A rodent model that exhibits all criteria of human DN has not yet been developed. However, the currently available rodent models of diabetes can be useful in the study of DN by increasing our understanding of the criteria of each diabetic rodent model [22].

### Experimental procedures

Twenty normal rats were randomly divided into two equal groups: the normal (*n* = 10 rats) and the UTI-treated control (*n* = 10 rats). The normal group received STZ vehicle (0.1 mol/l citrate buffer, pH 4.5) intraperitoneally, while the UTI-treated control group received UTI (100,000 U/kg) daily intraperitoneally. Furthermore, twenty diabetic rats were randomly divided into two equal groups: the untreated DN (*n* = 10 rats) and the UTI-treated DN (*n* = 10 rats). The untreated DN group received 0.9% saline intraperitoneally in the same volume as the UTI-treated control group, while the UTI-treated DN group received the same dose of UTI as the UTI-treated control group. All groups received the treatment for four weeks [43].

### Blood and tissue collection

At the end of the experiment, rats were anesthetized with an i.p injection of sodium pentobarbital (60 mg/kg) [33] and sacrificed. The serum was separated from the blood samples by centrifugation at 4000 × g for 15 min and stored at − 80 °C until used in biochemical assays. All rat kidneys were immediately dissected, weighed, and washed in ice-cold saline. One kidney from each rat was stored in 10% neutral buffered formalin for histopathological and immunohistochemical (IHC) analysis. The other kidney was immediately frozen at − 80 °C until tissue homogenization, mitochondria separation, and RNA extraction. Furthermore, 7 cm of ileum was excised from rats, and parts of the ileal tissues were used for tissue homogenization and biochemical analysis, while other parts were fixed in 10% neutral buffered formalin for histological and IHC studies.

### Preparation of tissue homogenates and subsamples

One piece of renal and intestinal tissues was homogenized at 10% (w/v) in 50 mM phosphate buffer (pH 7.4). After centrifuging the homogenate at 5000 × g for 20 min at 4 °C, the supernatant was aliquoted and kept at − 80 °C until use. Another piece of renal tissue was homogenized on ice in mitochondrial isolation buffer (0.01 M Tris–HCl, 0.0001 M EDTA-2Na, 0.01 M sucrose, 0.8% NaCl, pH 7.4) using a Teflon Potter homogenizer. The homogenate was centrifuged at 1500 rpm for 5 min at 4 °C. The supernatant was centrifuged again at 10,000 rpm for 15 min. The precipitate obtained represented the mitochondria fraction [12].Total protein content was measured in renal and intestinal tissue homogenates as well as renal mitochondrial subsamples using the method of Lowry et al. [26].

### Assessment of STZ-induced diabetic state

The serum FBG level was measured using a colorimetric commercial kit (Spinreact, Barcelona, Spain, catalog no. MDBSIS46). In addition, the serum insulin level was measured using an enzyme linked immunosorbent assay (ELISA) kit (Fine Biotech, Wuhan, China, catalog no. ER1113).

### Assessment of STZ-induced DN

The blood urea nitrogen (BUN) levels, as well as serum and urinary creatinine levels, were colorimetrically measured using kits purchased from Biodiagnostic (Giza, Egypt, catalog nos. UR 21 10 and CR 12 50, respectively). Furthermore, urinary albumin levels were measured using an immunoturbidmetric method (BioSystems, Barcelona, Spain, catalog no. 13324) according to the manufacturer’s protocol. Finally, creatinine clearance was calculated using the following formula:

$$Creatinine\;clearance\;(ml/min)=urine\;creatinine\;(mg/dl)\times urine\;volume\;(ml/24\;hours)/serum\;creatinine\;(mg/dl)\times1440$$ [6] 

### Assessment of serum Complement 5a (C5a) and short chain fatty acid (SCFA) levels

The serum C5a and SCFA levels were measured using ELISA kits (MyBiosource, San Diego, USA, catalog no. MBS268981 and MBS7269667, respectively) according to the manufacturers’ protocol.

### Assessment of renal and intestinal inflammatory markers

Nuclear factor kappa B (NF-κB) levels in renal and intestinal tissue homogenates, as well as renal monocyte chemoattractant protein-1 (MCP-1) levels, were determined using ELISA kits (MyBiosource, San Diego, USA, catalog no. MBS453975 and MBS2506535, respectively) according to the manufacturers’ protocol.

### Assessment of renal intercellular adhesion molecule 1 (ICAM1) and signal transducer and activator of transcription 3 (STAT3) mRNA expression levels by real-time PCR

Total RNA was isolated from kidney samples using the GeneJET RNA Purification Kit (Thermo Scientific, USA, catalog no. K0731). Total RNA (5 μg) was then reverse transcribed into cDNA with Revert Aid H Minus Reverse Transcriptase (Thermo Scientific, USA, catalog no. EP0451). The cDNA was used as a template to determine the relative expression of renal *ICAM1* and *STAT3* genes using Step One Plus real time PCR system (Applied Biosystem, USA), with *β-actin* serving as a reference gene [7]. The primers sequences were as follows: *ICAM1*, forward (5′-GCTTCTGCCACCATCACTGTGTA-3′); reverse (5′-ATGAGGTTCTTGCCCACCTG-3′); *STAT3*, forward (5′-CACCCATAGTGAGCCCTTGGA-3'); reverse (5′-TGAGTGCAGTGACCAGGACAGA-3'); *β-actin*, forward (5'-GGAGATTACTGCCCTGGCTCCTA-3'); reverse (5'-GACTCATCGTACTCCTGCTTGCTG-3'). The relative gene expression was calculated using the 2^−∆∆Ct^ method [25].

### Assessment of renal mitochondrial parameters

Mitochondrial reactive oxygen species (ROS) was evaluated in the mitochondria subsamples following the manufacturer’s instructions using spectrophotometric kit provided by Cayman Chemical Company (Ann Arbor, MI, USA; catalog no. 701600). Dynamin-related protein 1 (Drp1) and mitochondrial fission 1 protein (FIS1) levels were assessed in mitochondria subsamples using ELISA kits (SunRed Biological Technology, Shanghai, China, catalog no. 201–11-3125, and MyBiosource, San Diego, USA, catalog no. MBS2890155, respectively) according to the manufacturer’s instructions. Mitochondrial ATP level were measured using a colorimetric ATP assay kit obtained from BioVision, CA, USA, (catalog no. K354-100) following the manufacturer’s instructions. Furthermore, mitochondrial transmembrane potential (ΔΨm) was assessed using methods of Maity et al. [28] and Guha et al. [15] using the cationic carbocyanine dye (JC-1) (HY-15534) (MedChemExpress, New Jersey, USA). In brief, 20 µg of freshly prepared isolated mitochondria were incubated in the dark with JC-1 for 10 min at 37 °C. The JC-1 dye exhibited a potential-dependent accumulation in the mitochondria indicated by a fluorescence emission shift from green at 529 nm wavelength to red at 590 nm wavelength. Therefore, a decrease in the red/green fluorescence intensity ratio was used to identify the ΔΨm. The concentration-dependent synthesis of red fluorescent J-aggregates (different aggregate formation rates) was the cause of the potential-sensitive colour shift.

### Assessment of renal oxidative stress markers

The hydrogen peroxide (H_2_O_2_) levels were measured colorimetrically using a commercial kit (Biodiagnostic, Giza, Egypt, catalog no. HP 25), where H_2_O_2_ reacts with 3,5-dichloro-2-hydroxybenzensulfonic acid and 4-aminophenazone in the presence of peroxidase to form a chromophore [3]. Furthermore, 8-hydroxy-2'-deoxyguanosine (8-OHdG) levels, a marker of oxidative DNA damage, were assayed using an ELISA kit (Fine Biotech, Wuhan, China, catalog no. ER1487) according to the manufacturer’s instructions.

### Histopathologic evaluation and quantitative morphometric analysis

Following the fixation of intestinal and kidney tissue samples, paraffin blocks were prepared. Ileal sections were stained with hematoxylin and eosin (H&E) and Periodic acid-Schiff (PAS) to demonstrate goblet cells. Kidney sections were stained with H&E and Masson trichrome (MT) stain to assess collagen deposition.

Images were captured with a Leica ICC50 digital camera connected to a Leica DM500 microscope and then analysed with the image analysis software Fiji (ImageJ bundled with plugins, National Institute of Mental Health, Bethesda, Maryland, USA). For intestinal sections, intestinal villus height was measured from the base of the villus to its tip, whereas crypt depth was measured from the base of the villus to the muscularis mucosae [8]. The number of goblet cells per crypt-villus unit (CVU) was counted on PAS-stained sections using a plugin cell counter with Fiji. In each animal, all measurements were taken in 20 randomly selected crypt-villus unit [31]. For kidney sections, the glomerular tuft area was measured in 10 randomly selected glomeruli per animal [36]. The percentage of MT-stained area was determined to assess the extent of collagen deposition [13].

### Intestinal claudin-1 and renal TGF-β1 immunohistochemical staining

The immunostaining was carried out using a Dako automated stainer (Link 48) applying the EnVision + Dual Link System- horseradish peroxidase (HRP). This system is based on an HRP labelled polymer which is conjugated with secondary antibodies. Sections on positive charge slides were deparaffinized, and then subjected to a high pH Envision TM FLEX Retrieval Solution at 97 °C for 20 min in DAKO PT Link unit. The slides were then incubated with primary antibodies for 30 min. The primary antibodies were claudin-1 rabbit polyclonal antibody (catalog no. RB-9209-R7, Thermo Fisher Scientific company, USA) for intestinal sections and TGF-β1 mouse monoclonal antibody (catalog no. MA1-34,093, Invitrogen, Thermo Fisher Scientific company, USA) for kidney sections. Slides were then incubated in HRP solution for 30 min. Diaminobenzidine (DAB) solution was applied as chromogen, and the slides were counterstained with hematoxylin. The staining intensity for intestinal claudin-1 and renal TGF-β1 was quantified using image analysis software (Fiji). Three random images from each slide were captured at × 200. The image was submitted to the plug-in "color deconvolution" using the built-in vector HDAB, where the hematoxylin and DAB staining were separated into three different panels (hematoxylin, DAB only image, and background). The DAB image demonstrating the stained areas was represented with the colour-2 image. Five regions of interest (ROI), demonstrating the brown staining, were identified. Then, we run analyse, set measurements, and select the “Mean grey value”. The final DAB intensity was calculated using the formula (f = 255 – i), where "f" is the final DAB intensity and "i" is the mean grey value obtained from the software, with 0 representing no stain and 255 representing the strongest stain. Finally, the mean values were calculated for each slide [1].

### Statistical analysis

The statistical analysis was performed using the Statistical Package for the Social Sciences (SPSS) software, version 20.0 (SPSS Inc., Chicago, IL, USA). All data were checked for normality using the Kolmogorov–Smirnov test. The collected data were subjected to one–way ANOVA followed by Tukey’s test that used to determine the significance between groups. Pearson’s correlations were used to correlate the measured markers of the gut axis and renal function tests in the UTI-treated DN group. The data were expressed as mean and standard deviation (SD). All data were derived from biological replicates. *P*-values of less than 0.05 were considered statistically significant.

## Results

### Effect of UTI treatment on biochemical parameters of DN

**Fig. 1:**
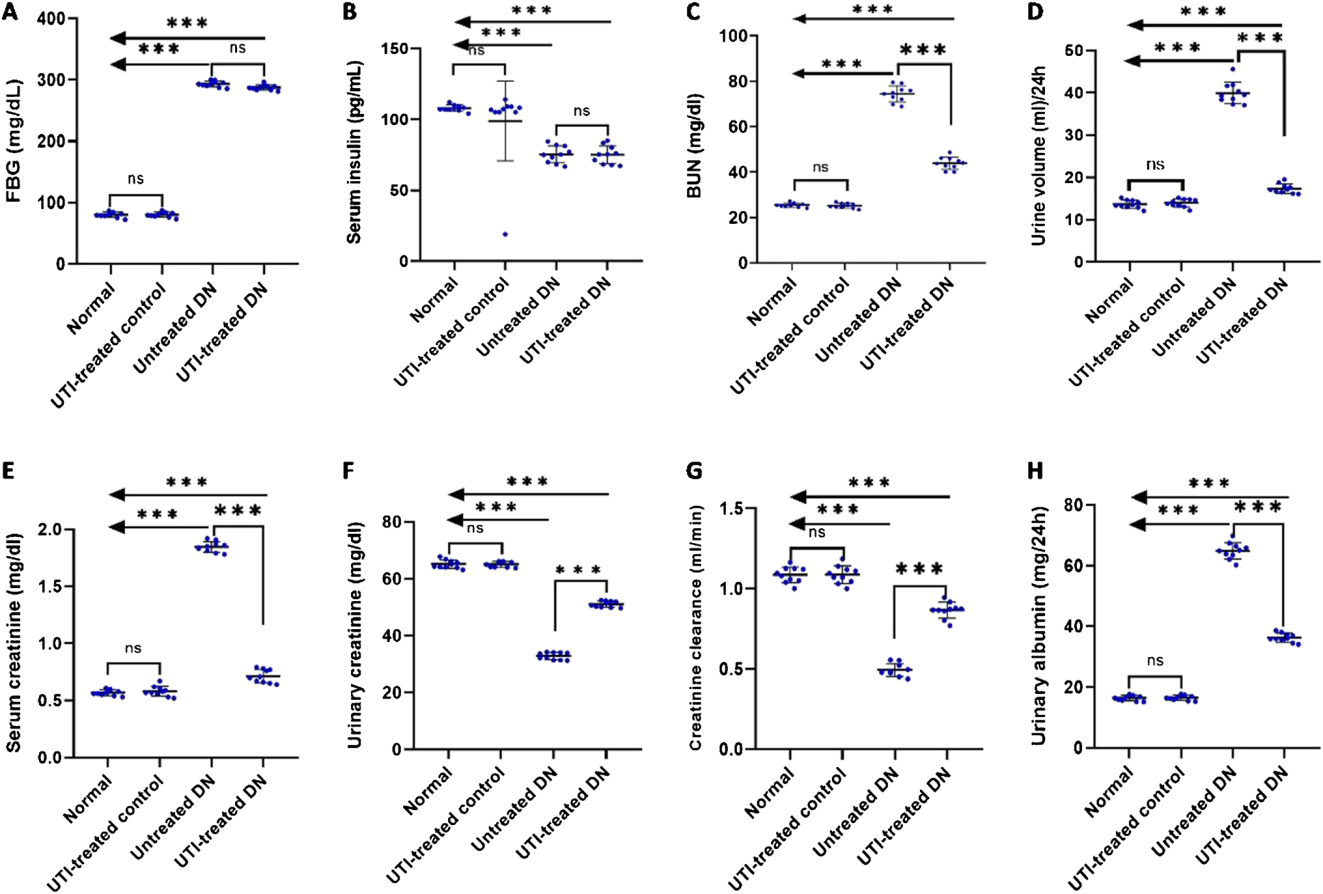
Effect of UTI treatment on various biochemical parameters in diabetic nephropathy (DN) (**A**) Fasting blood glucose (FBG) levels, (**B**) serum insulin levels, (**C**) blood urea nitrogen (BUN) levels, (**D**) urine volume (ml)/24 h (**E**) serum creatinine levels, (**F**) urinary creatinine levels, (**G**) creatinine clearance, and (H) urinary albumin levels. FBG, BUN, and serum and urinary creatinine levels were colorimetrically assayed, while serum insulin levels were assayed by ELISA. Data are expressed as mean ± SD, *n* = 10 rats for each group, ns, not significant and ^***^ indicates *p* < 0.001 using one–way analysis of variance followed by Tukey’s test

UTI treatment for 4 weeks significantly reduced BUN, urine volume/24 h, serum creatinine, and urinary albumin in the UTI-treated DN group compared to the untreated DN group. Moreover, it increased urinary creatinine and creatinine clearance in the UTI-treated DN group compared to the untreated DN group, with no significant differences in FBG and serum insulin levels. Furthermore, none of these parameters differed significantly after UTI treatment in the UTI-treated control group compared to the normal group (Fig. 1).

### Effect of UTI treatment on the components of the gut-kidney axis

#### Morphological changes in the ileum

**Fig. 2:**
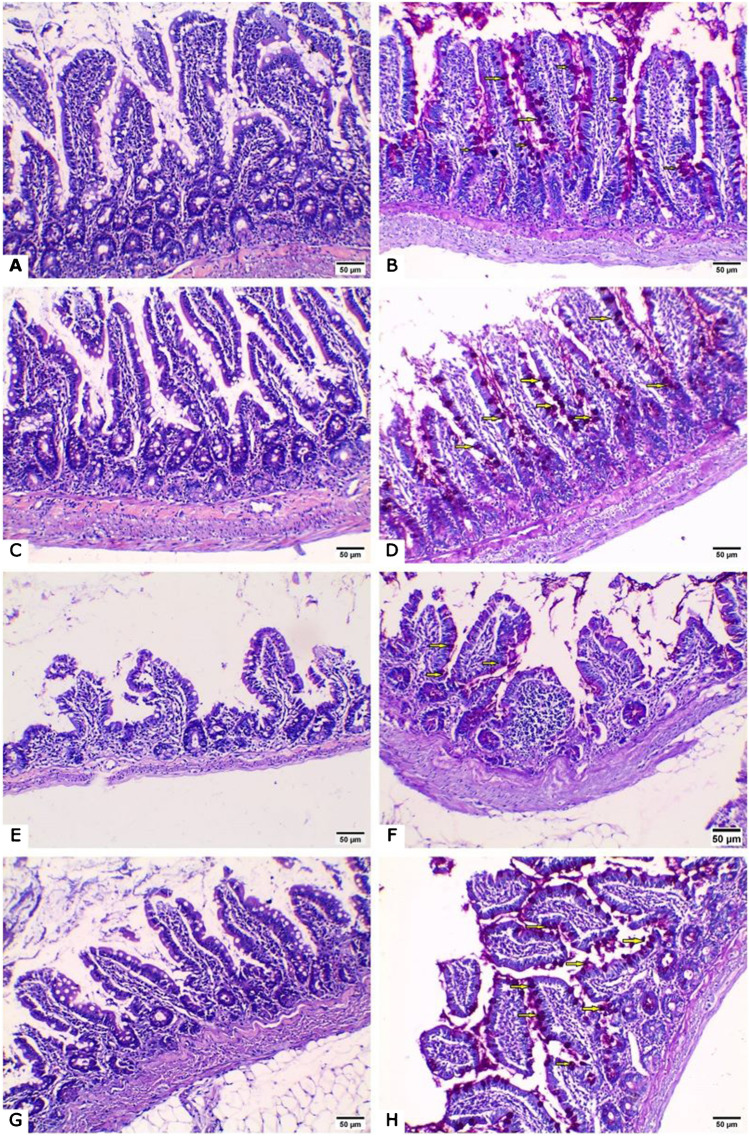
Hematoxylin and eosin-and Periodic acid-Schiff-stained intestinal sections (A, B) Normal group with (**A**) the intestinal wall showing normal arrangement of intestinal villi and crypts and (**B**) a large number of PAS-positive goblet cells scattered between the intestinal epithelial cells (yellow arrows). (**C**, **D**) The UTI-treated control group, where the intestinal mucosa is similar to that of the normal control group. (E, F) The untreated DN group showing (**E**) intestinal villous atrophy with prominent decrease in the villus height as well as crypt depth, and (**F**) a marked decrease in the number of PAS-positive goblet cells (yellow arrows) with prominent inflammatory cellular infiltrate within the villus core. (G, H) The UTI-treated DN group showing **G**) improvement of the intestinal lining as most villi have returned to their normal pattern with minimal inflammatory infiltrate, and (**H**) an increase in the number of PAS-positive goblet cells (yellow arrows). Hematoxylin and eosin staining (A, C, E, G) and Periodic acid-Schiff staining (B, D, F, H) (× 200, scale bar = 50 μm)

**Fig. 3:**
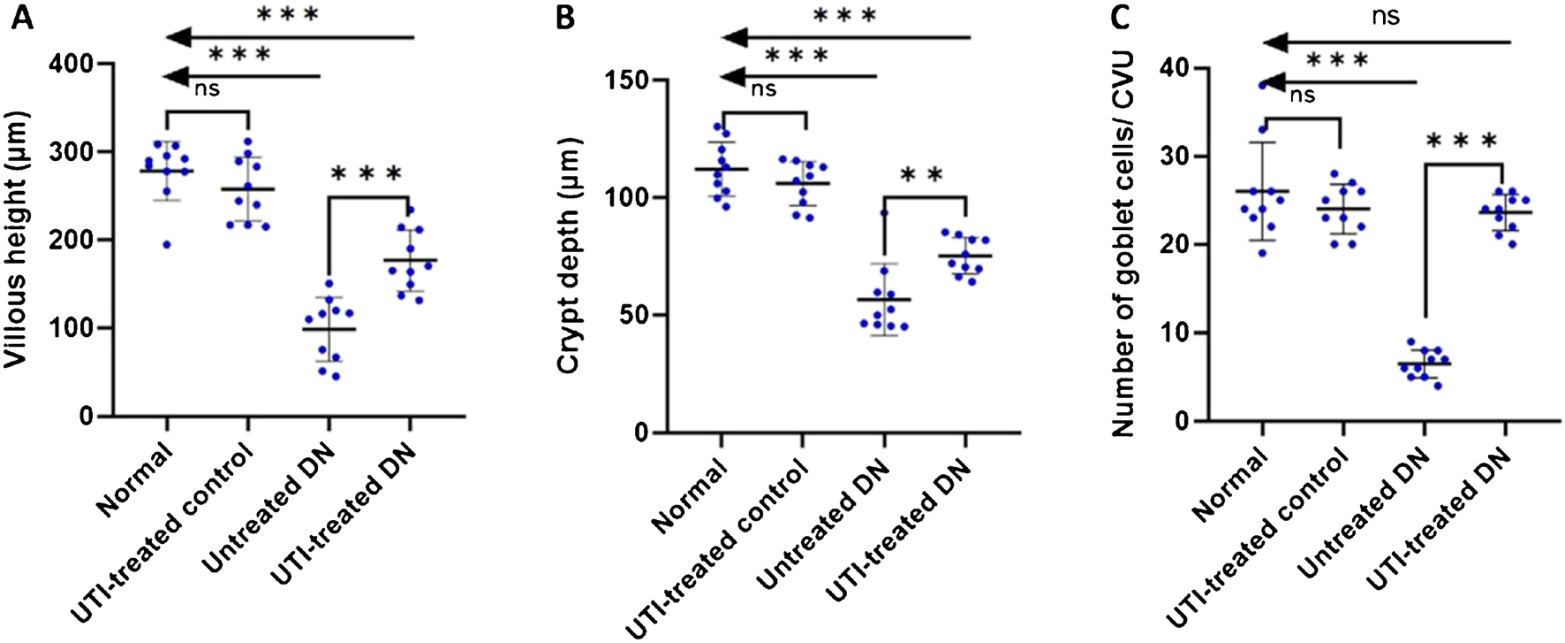
Morphometric analysis of the (**A**) villus height (µm), (**B**) crypt depth (µm), and (**C**) the number of goblet cells/CVU. All data are expressed as mean ± SD; *n* = 10 rats for each group; DN, diabetic nephropathy; ns, not significant, ^**^ indicates *p* < 0.01 and ^***^ indicates *p* < 0.001 using one–way analysis of variance followed by Tukey’s test

H&E-stained ileal sections from normal and UTI-treated control groups revealed normally organized villi and crypts with intact epithelial covering (Fig. 2A, C). PAS-stained sections revealed numerous goblets cells scattered among the enterocytes (Fig. 2B, D). On the other hand, sections from the untreated DN group revealed areas of epithelial erosion and marked inflammatory cellular infiltration (Fig. 2E, E). UTI treatment of the DN group markedly improved the intestinal changes, as most villi and crypts restored their normal architecture with minimal inflammatory infiltrate (Fig. 2G, H). Morphometric analysis of the villus height, crypt depth, and number of goblet cells/CVU in PAS-stained sections revealed no significant difference between the normal and UTI-treated control groups. However, there was a significant reduction in villus height, crypt depth and the number of goblet cells/CVU in the untreated DN group compared to the normal group. When compared to untreated DN group, UTI treatment of the DN group significantly increased villus height, crypt depth, and the number of goblet cells/CVU. Furthermore, the number of goblet cells/CVU in the UTI-treated DN group was nearly equal to that in the normal group (Fig. 3).

### UTI treatment improved claudin-1 expression in the intestine of DN rats

**Fig. 4:**
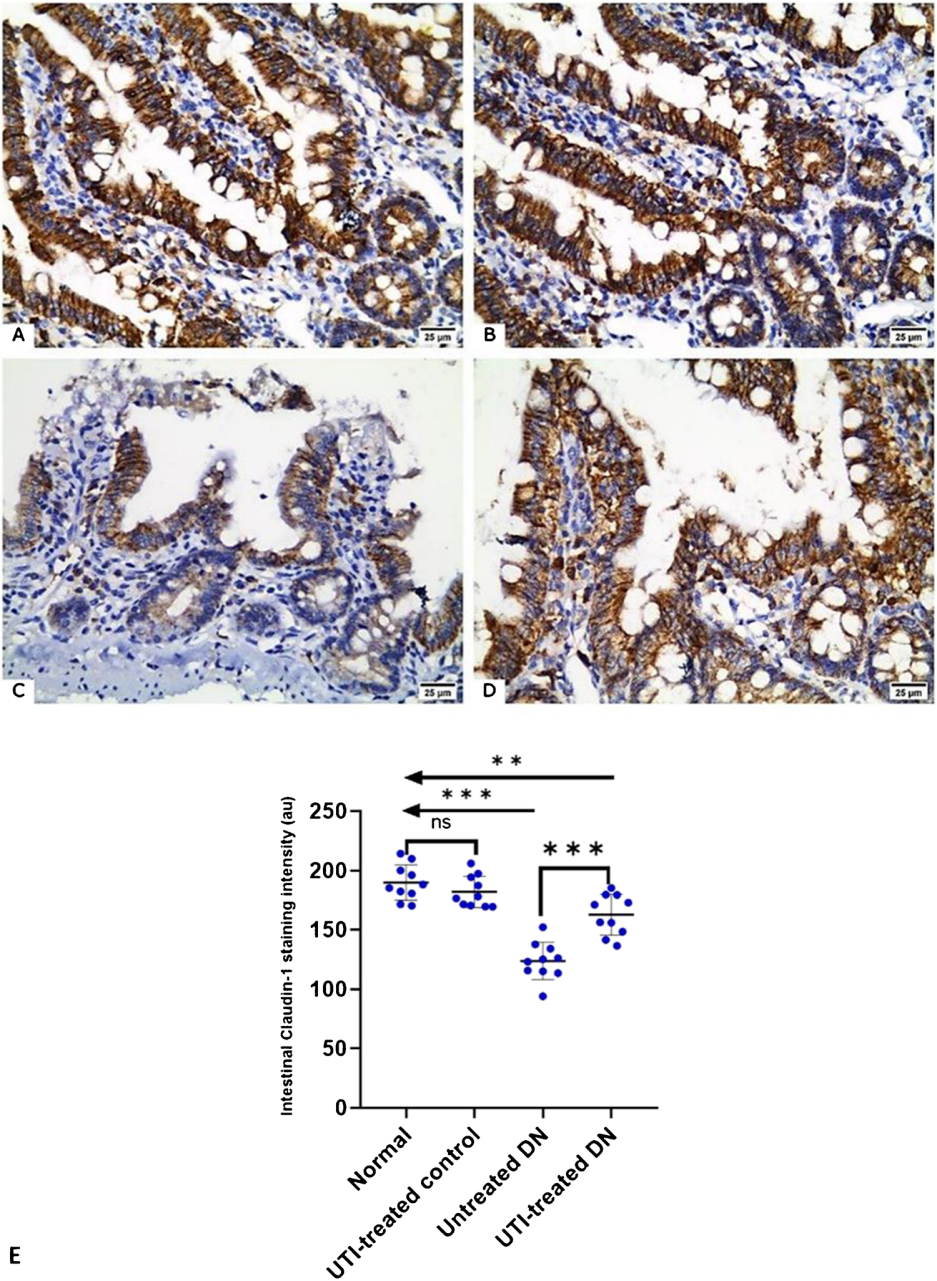
Claudin-1 immunostained intestinal sections from the different studied groups Claudin-1 expression was detected as brownish cytoplasmic staining in the epithelial lining of the intestinal villi and crypts. Claudin-1 staining in (**A**) the normal group, (B) UTI-treated control group, (**C**) untreated DN group, and (**D**) UTI-treated DN group (× 400, scale bar = 25 μm). (E) Morphometric analysis of intestinal Claudin-1 staining intensity. All data are expressed as mean ± SD; *n* = 10 rats for each group; a.u., arbitrary unit; DN, diabetic nephropathy; ns, not significant. ^**^ indicates *p* < 0. 01 and ^***^ indicates *p* < 0.001 using one–way analysis of variance, followed by Tukey’s test

UTI treatment increased the expression of intestinal tight-junction protein. Claudin-1 expression was highest in the normal and UTI-treated control groups, with no significant difference between the two groups. It was significantly lower in the untreated DN group compared to the normal group. However, UTI treatment restored the reduced intestinal expression of claudin-1 in the UTI-treated DN group compared to untreated DN group (Fig. 4).

### UTI treatment ameliorated intestinal, serum, and renal inflammatory markers

**Fig. 5:**
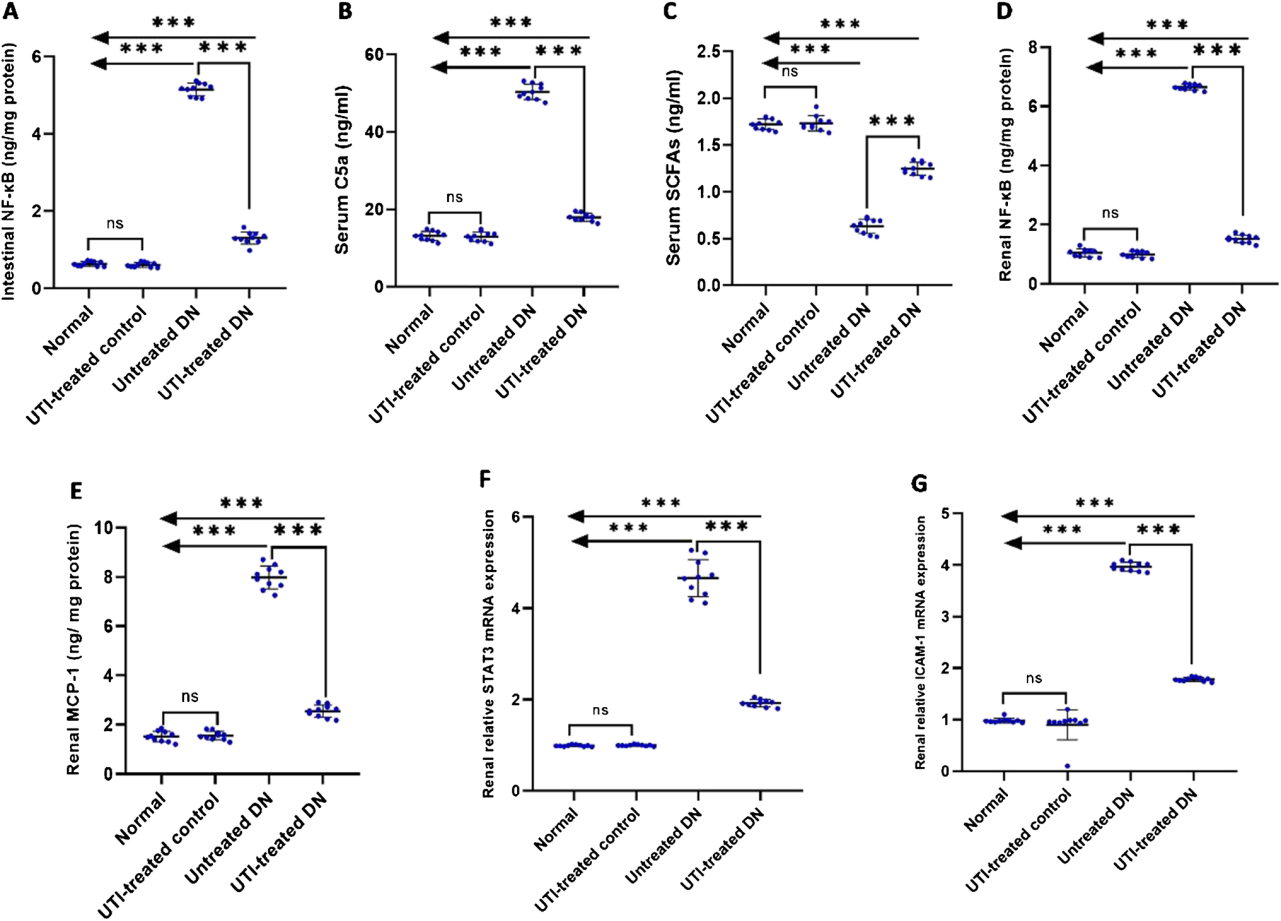
UTI treatment ameliorated intestinal, serum, and renal inflammatory markers (**A**) Intestinal NF-κB levels, (**B**) serum C5a levels, (**C**) serum SCFA levels, (**D**) renal NF-κB levels, (**E**) renal MCP-1 levels, (**F**) renal relative *STAT3* mRNA expression, and (**G**) renal relative *ICAM-1* mRNA expression. Intestinal NF-Κb, serum C5a and SCFA, and renal NF-κB and MCP-1 levels were determined using ELISA, while relative expressions of renal *STAT3* and *ICAM-1* mRNA were assessed using real-time PCR. Data are expressed as mean ± SD, *n* = 10 rats for each group; DN, diabetic nephropathy; NF-κB, nuclear factor kappa B, C5a, complement 5a; SCFAs, short chain fatty acids; MCP-1, Monocyte chemoattractant protein-1; STAT3, signal transducer and activator of transcription 3 factor; ICAM-1, intercellular adhesion molecule 1; ns, not significant. ^***^ indicates *p* < 0.001 using one–way analysis of variance, followed by Tukey’s test

UTI treatment significantly reduced the levels of intestinal and renal NF-κB, serum C5a, and renal MCP-1 in the UTI-treated DN group compared to the untreated DN group. In addition, it downregulated the renal *STAT3* and *ICAM-1* expression in the UTI-treated DN group compared to the untreated DN group. On the other hand, it significantly increased serum SCFA levels in the UTI-treated DN group compared to the untreated DN group. However, UTI had no effects on any of the aforementioned parameters in the UTI-treated control group when compared to the normal group (Fig. 5).

### UTI treatment reduced glomerular injury and collagen deposition in the kidneys of DN rats

**Fig. 6:**
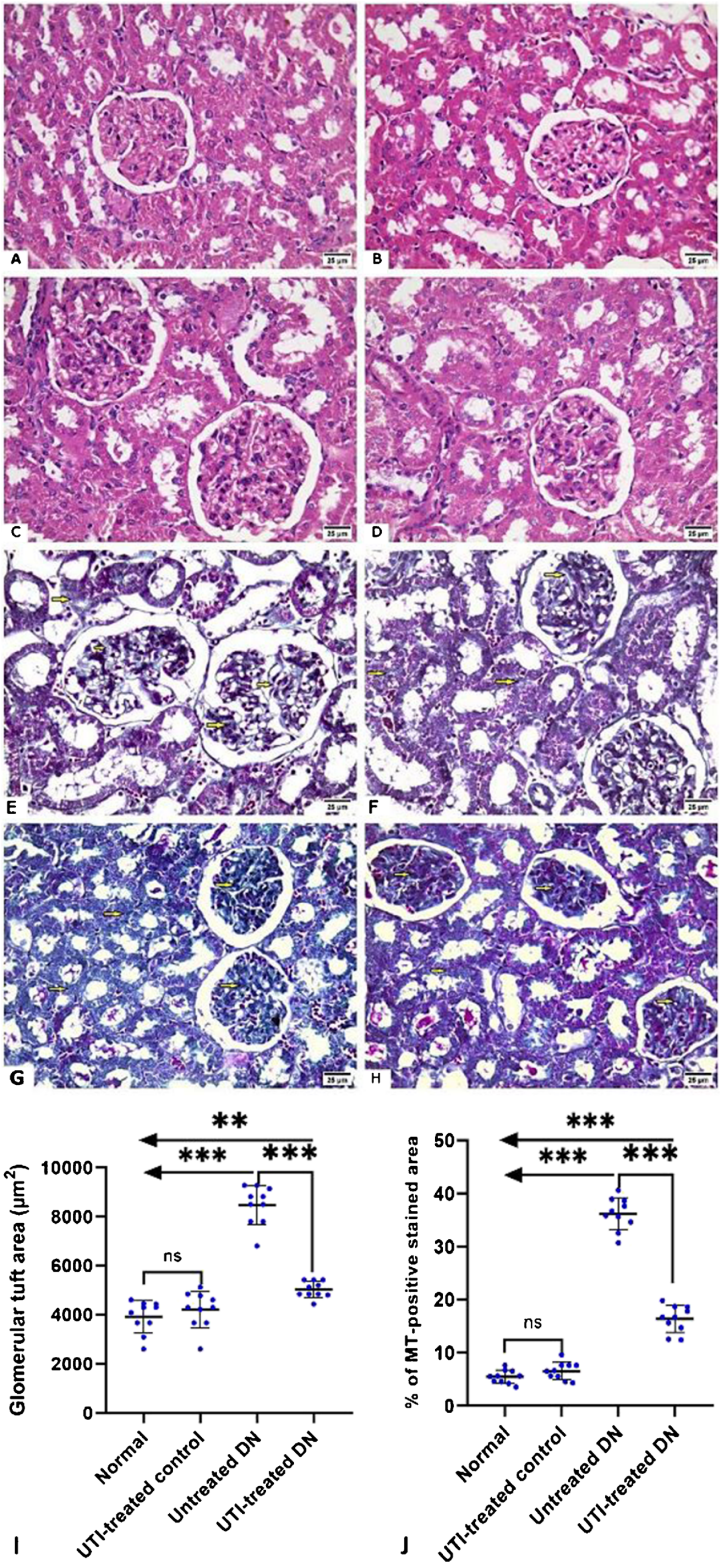
Hematoxylin and eosin-and Masson trichrome-stained kidney sections from the different studied groups (**A**) The normal and (**B**) UTI-treated control groups showing normal appearance of the glomeruli and tubules. (**C**) The untreated DN group showing prominent mesangial hypercellularity with increased glomerular tuft area, and capillary congestion. (**D**) The UTI-treated DN group showing that the glomeruli and tubules regained its normal appearance. (**E**, **F**) The normal and UTI-treated control groups showing minimal insignificant deposition of collagen within the glomeruli (yellow arrows). (**G**) The untreated DN group showing marked collagen deposition with accumulation within the glomeruli and between the tubules (yellow arrows). (**H**) The UTI-treated DN group showing that UTI treatment reduced in the percentage of collagen deposition (yellow arrows). (**I**, **J**) Morphometric analysis of glomerular tuft area (µm2) and percentage of MT-positive stained area, respectively. All data are expressed as mean ± SD; *n* = 10 rats for each group; ns, not significant. ^**^ indicates *p* < 0.01 and.^***^ indicates *p* < 0.001. H&E, hematoxylin and eosin; MT, Masson trichrome; DN, diabetic nephropathy. H& E staining (A, B, C, D) and MT staining (E, F, G, H) (× 400, scale bar = 25 μm)

H&E-and MT-stained kidney sections from the normal group revealed normal architecture of the glomeruli and tubules, with minimal collagen deposition in the MT-stained sections. Furthermore, sections from the UTI-treated control group were similar to those from the normal group, with no significant difference in glomerular tuft area or amount of deposited collagen between the two groups. On the other hand, untreated DN group sections revealed mesangial hypercellularity with a significant increase in glomerular tuft area and prominent periglomerular and peritubular collagen deposition when compared to the normal group. UTI treatment improved the kidney changes in the UTI-treated DN by significantly reducing glomerular tuft area and periglomerular and peritubular collagen deposition when compared to the untreated DN group (Fig. 6).

### UTI treatment reduced tubular TGF-β1 expression in the kidneys of DN rats

**Fig. 7:**
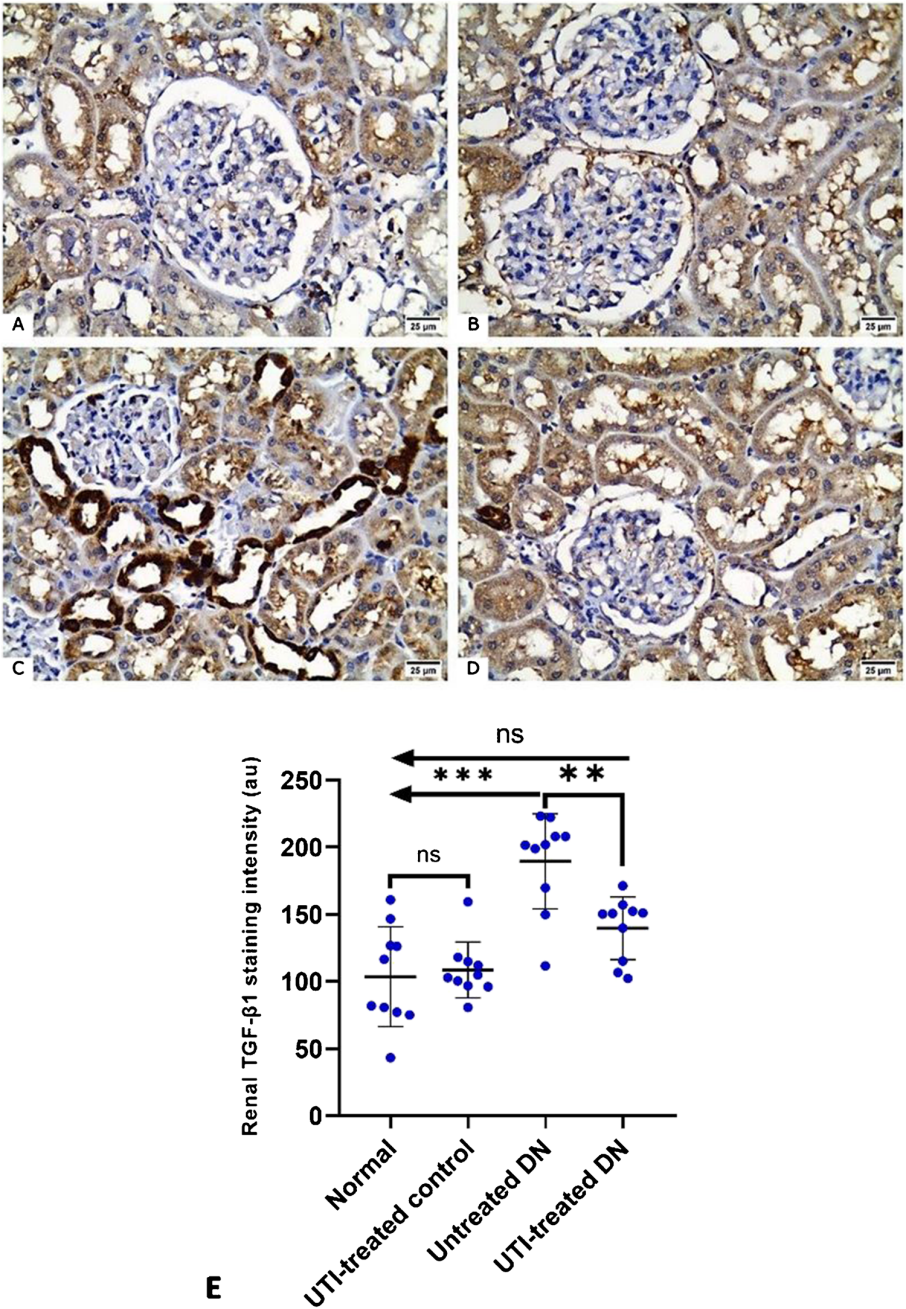
Transforming growth factor-beta 1 (TGF-β1) immunostained kidney sections from the different studied groups (**A**, **B**) The normal and UTI-treated control groups, respectively, (**C**) the untreated DN group, and (**D**) the UTI-treated DN group. (× 400, scale bar = 25 μm). (**E**) Morphometric analysis of renal TGF-β1 staining intensity. All data are expressed as mean ± SD; *n* = 10 rats for each group; a.u., arbitrary unit; DN, diabetic nephropathy; ns, not significant. ^**^ indicates *p* < 0.01 and ^***^ indicates *p* < 0.001 using one–way analysis of variance, followed by Tukey’s test

TGF-β1 expression was detected as brownish staining in the cytoplasm of tubular epithelial cells. TGF-β1 staining was mild in both the normal and UTI-treated control groups. Furthermore, when compared to the normal group, the untreated DN group had significantly higher TGF-β1 staining intensity in the tubules and a few cells within the glomeruli. However, UTI treatment significantly ameliorated this effect in the UTI-treated DN group compared to the untreated DN group, with no significant difference between the UTI-treated DN group and the normal group (Fig. 7).

### UTI treatment showed potential beneficial effects on mitochondrial function and redox equilibrium

**Fig. 8:**
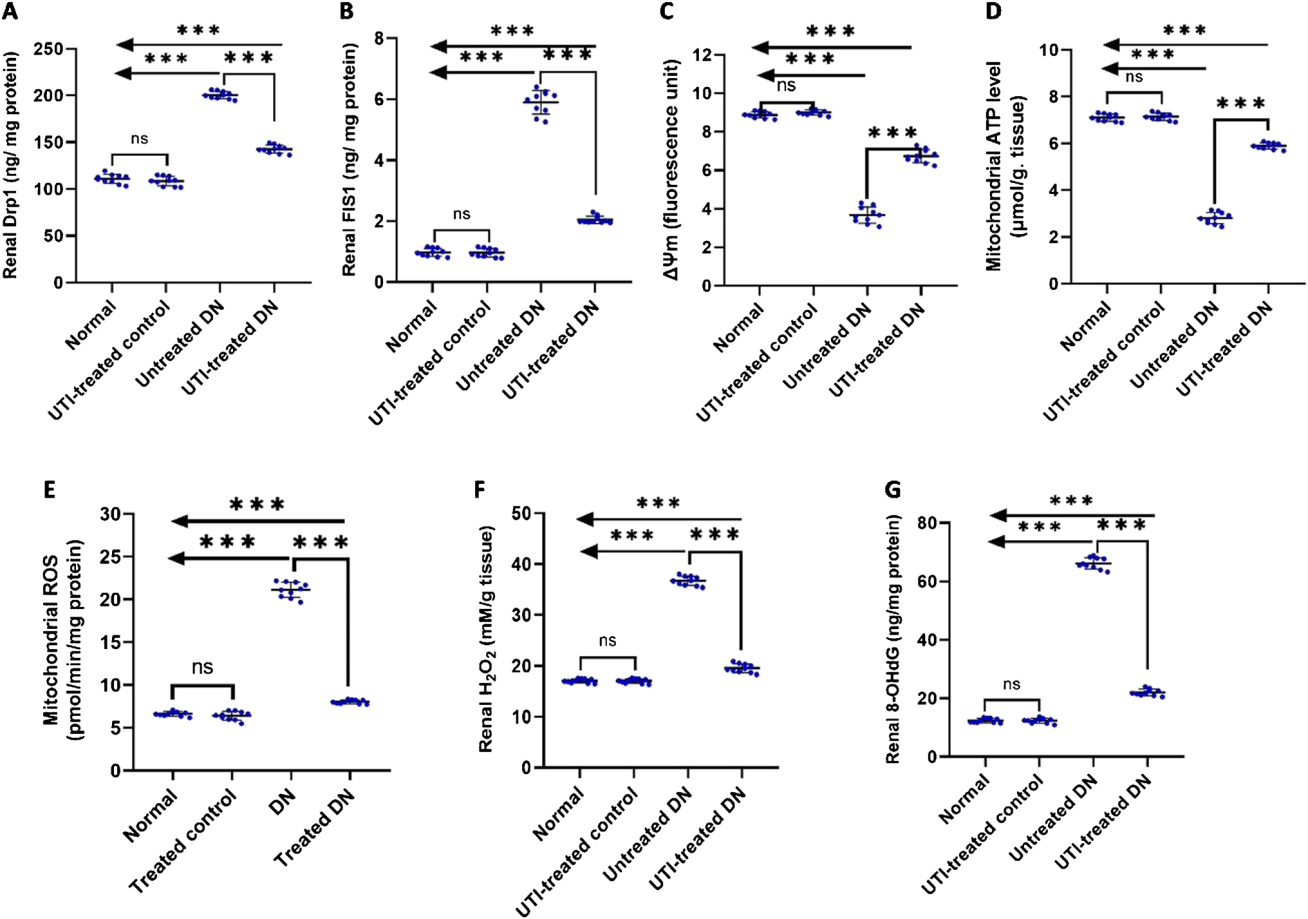
UTI treatment improved renal mitochondrial functions and redox equilibrium (**A**) Dynamin-related protein 1 (Drp1) levels, (**B**) mitochondrial fission 1 protein (FIS1) levels, (**C**) mitochondrial transmembrane potential (ΔΨm), (**D**) mitochondrial ATP level, (**E**) mitochondrial ROS levels, (**F**) hydrogen peroxide (H_2_O_2_) levels, and (**G**) 8-hydroxy-2'-deoxyguanosine (8-OHdG) levels. Drp1and FIS1 levels were assessed in mitochondrial subsamples using ELISA, while mitochondrial ATP and H_2_O_2_ levels were measured colorimetrically. Furthermore, ΔΨm levels were assessed using the cationic carbocyanine dye (JC-1) (HY-15534), while 8-OHdG levels were assayed using ELISA. Data are expressed as mean ± SD; *n* = 10 rats for each group; DN, diabetic nephropathy; ns, not significant. ^***^ indicates *p* < 0.001 using one–way analysis of variance, followed by Tukey’s test

UTI treatment for four weeks resulted in significant reductions in mitochondrial fission proteins, including Drp1and FIS1, as well as mitochondrial ROS levels while it significantly increased mitochondrial ATP level and mitochondrial ΔΨm in the UTI-treated DN group compared to the untreated DN group. Furthermore, it significantly reduced oxidative stress markers such as H_2_O_2_ and 8-OHdG in the UTI-treated DN group when compared to the untreated DN group. However, UTI has no effect on these parameters in the UTI-treated control group when compared to the normal group (Fig. 8).

### Correlation between C5a, claudin-1, SCFAs, intestinal NF-κB, and renal function tests in UTI-treated DN group

**Fig. 9:**
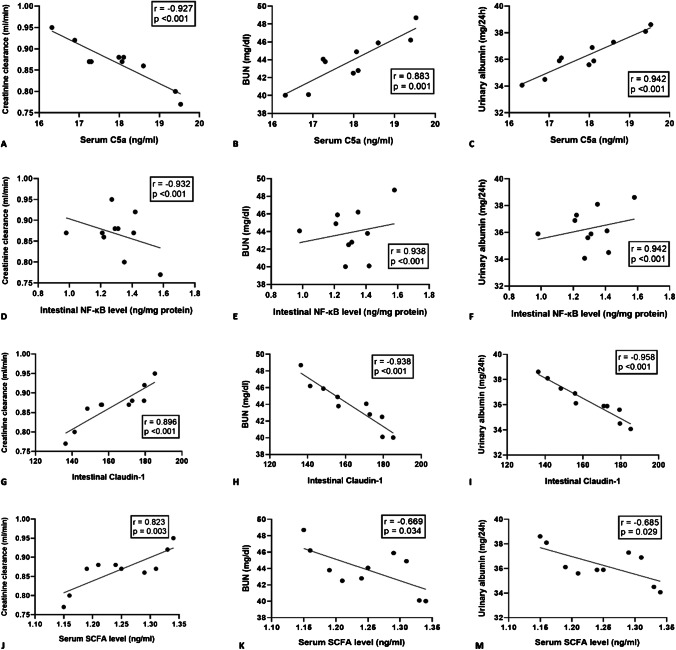
Correlations between C5a, claudin-1, SCFAs, intestinal NF-κB, and renal function tests in UTI-treated DN group. UTI-treated DN, Ulinastatin-treated diabetic nephropathy; C5a, Complement 5a; SCFAs, short chain fatty acids; NF-κB, Nuclear factor kappa B, BUN, blood urea nitrogen. *n* = 10 rats for each group. *P*-values of less than 0.05 were considered statistically significant. Pearson’s correlations were used to correlate the measured markers of the gut axis and renal function tests in the UTI-treated DN group

Pearson’s correlations analysis showed that serum C5a and Intestinal NF-κB were negatively correlated with creatinine clearance while they positively correlated with BUN and urinary albumin in UTI-treated DN group (Fig. 9A-F). On the other hand, intestinal claudin-1 and SCFAs were positively correlated with creatinine clearance while they negatively correlated with BUN and urinary albumin in UTI-treated DN group (Fig. 9G-M).

## Discussion

The present study demonstrated for the first time the effect of UTI treatment on DN. It could ameliorate DN as it significantly improved kidney functions markers such as BUN, serum creatinine, urine volume/24 h, urinary albumin, and creatinine clearance. Furthermore, it improved the histological changes in the kidneys of the DN group. However, when compared to the DN group, UTI treatment had no significant effect on FBG and serum insulin levels in the treated DN group.

The effect of UTI on kidney functions was consistent with a previous study, which found that UTI treatment improved kidney functions in patients with acute kidney injury induced by severe sepsis by significantly lowering BUN and serum creatinine levels [14]. Previous research on the effect of UTI on blood glucose levels has yielded conflicting results. Sharma et al. found that UTI had no significant effect on hyperglycemia in STZ-induced DM [32], whereas Zhao et al. found that UTI administration improves stress-induced hyperglycemia by reducing inflammation and improving insulin resistance [46]. These controversies could be explained by different models used in different studies.

The beneficial effects of UTI in DN could be attributed to its effect on the components of gut-kidney axis as indicated by significant correlations between measured markers of the components of gut-kidney axis and renal function tests in UTI-treated DN group. Moreover, the present study’s findings revealed that UTI treatment could significantly improve intestinal barrier integrity, and reduce intestinal and renal inflammation by increasing the expression of intestinal tight junction protein claudin-1, villus height, crypt depth, the number of goblet cells/CVU, and serum SCFA levels while decreasing the levels of inflammatory markers in the serum, intestine, and kidneys, such as serum C5a, intestinal NF-κB, renal NF-κB, and renal MCP-1, as well as renal STAT3, renal ICAM-1, and renal TGF-β1 expression.

Growing evidence suggests that increased intestinal permeability in DM may be due to complement system activation, including the formation of C3a and C5a, as a result of C3 and C5 cleavage. Then, C3a and C5a increase interleukin-8 production and activate the extracellular signal‑regulated kinase (ERK) pathway, resulting in increased intestinal permeability [10] and a decrease in intestinal tight junction proteins, including claudin-1, which is partially restored by a C5a receptor antagonist [23].

Moreover, C5 activation in DM promotes oxidative stress and kidney damage by increasing fibrogenic and inflammatory factor expression in renal tissues [42]. The up-regulated C5a activates STAT3, up-regulating cytokine expression and enhancing extracellular matrix synthesis, and thereby promoting inflammatory response and fibrosis in kidneys at early DN stages.

Accumulating data have emphasized that leucocyte recruitment is important in DN pathogenesis. Leukocyte infiltration is mediated by many types of adhesion molecules and chemokines. Intercellular adhesion molecule-1 (ICAM-1) and vascular cell adhesion molecule-1 (VCAM-1) promote leukocyte-endothelial cell adhesion; whereas MCP-1 promotes leucocyte migration from the vascular lumen to inflammatory sites. These proinflammatory molecules could be used as new therapeutic targets for DN [35].

In addition, elevated C5a induces gut microbiota dysbiosis, which reduces gut SCFA production, impairing the anti-inflammatory effects of endogenous SCFAs [23]. The anti-inflammatory effects of SCFAs prevent kidney injury by lowering reactive oxygen species (ROS) and cytokine production while boosting mitochondria biogenesis [5]. Exogenous SCFAs have previously been shown to improve insulin resistance, inhibit hyperglycemia-induced oxidative stress, prevent DN manifestations such as proteinuria and increased serum creatinine, inhibit NF-κB activation, suppress inflammatory cytokine, MCP-1, and interleukin-1β expressions, and prevent mesangial matrix accumulation and renal fibrosis [18] by suppressing hyperglycaemia-induced TGF-β1 synthesis [30]. These beneficial effects of increased SCFAs with UTI treatment on the DN group could explain the significant decreases in glomerular tuft area, mesangial hypercellularity, and capillary congestion in the treated DN group in this experiment.

TGF-β1 plays an active role in the pathogenesis of DN by increasing the synthesis of extracellular matrix proteins such as collagen IV, which accelerates renal tubulointerstitial fibrosis [29]. In this study, MT staining of the kidney and morphometric analysis of the percent of MT-positive stained area revealed that UTI treatment of the DN group resulted in decreased collagen deposition.

Furthermore, hyperglycemia in DM enhances ROS production [16], which induces chronic inflammation of the kidney with subsequent glomerular and tubular hypertrophy [19].

In this study, the effect of UTI on redox equilibrium was investigated for the first time. UTI improved the renal redox equilibrium and mitochondrial functions in the UTI-treated DN group, as evidenced by significant decreases in mitochondrial ROS, H_2_O_2_ and 8-OHdG and mitochondrial fission proteins, including Drp1 and FIS1, while it significantly increased mitochondrial ATP level and mitochondrial ΔΨm.

The kidney tissue is more vulnerable to oxidative stress because of the abundance of mitochondria in this tissue [37]. The DN activation of the complement system will disrupt the mitochondrial functions. The mitochondrial respiratory chain is the main site where ROS can be generated [11]. Previous studies have demonstrated that DM alters mitochondrial dynamics and increases mitochondrial fission, which alters the spatial orientation of the electron transport chain enzymes, promoting uncoupled respiration and ROS generation [44].

Mitochondrial fission is a leading cause of endothelial dysfunction in DM due to increased mitochondrial ROS, which leads to endothelial nitric oxide synthase uncoupling, cofactor oxidation, and target enzyme oxidative modification [38]. Previous studies reported that silencing FIS1 or Drp1 expression prevents hyperglycemia-induced ROS production and network fragmentation in endothelial cells. Therefore, pharmacological mitochondrial fission inhibitors are considered promising therapies for diabetic vascular disease [34].

There are some limitations to this study. First, the MT stain was only used to detect collagen deposition. It was, however, preferable to evaluate collagen IV immunohistochemically. Second, the study did not assess the effect of UTI on mitochondrial structure or pro-fusion markers. Third, because mitochondria were isolated from whole kidney samples, it is unclear from which cell type they originated. Finally, JC-1 was only a semi-quantitative readout of mitochondrial membrane potential.

## Conclusion

UTI treatment has the potential to slow the progression of DN possibly by modulating the components of the gut-kidney axis via improving intestinal barrier integrity and suppressing serum, intestinal, and renal inflammation, as well as improving renal mitochondrial functions and redox equilibrium. Future studies are required to gain detailed mechanistic insight into the effects of UTI on the gut-kidney axis and mitochondrial structure before this treatment can be implemented on a human scale.

## Data Availability

All data generated or analysed during this study are included in this published article.

## References

[1] Abdel Kerim AA, Ellity MM, El-Shorbagy SH, El-Guindy DM (2019). Cancer-associated fibroblasts in epithelial ovarian carcinoma: relation to clinicopathologic characteristics and microvessel density. Egypt J Pathol.

[2] Abo El Gheit R, Emam MN (2016). Targeting heme oxygenase-1 in early diabetic nephropathy in streptozotocin-induced diabetic rats. Physiol Int.

[3] Aebi H (1984). Catalase in vitro. Methods Enzymol.

[4] Alicic RZ, Rooney MT, Tuttle KR (2017). Diabetic Kidney Disease: Challenges, Progress, and Possibilities. Clin J Am Soc Nephrol.

[5] Andrade-Oliveira V, Amano MT, Correa-Costa M, Castoldi A, Felizardo RJ, de Almeida DC, Bassi EJ, Moraes-Vieira PM, Hiyane MI, Rodas AC, Peron JP, Aguiar CF, Reis MA, Ribeiro WR, Valduga CJ, Curi R, Vinolo MA, Ferreira CM, Camara NO (2015). Gut Bacteria Products Prevent AKI Induced by Ischemia-Reperfusion. J Am Soc Nephrol.

[6] Bazzano T, Restel TI, Porfirio LC, Souza AS, Silva IS (2015). Renal biomarkers of male and female Wistar rats (Rattus norvegicus) undergoing renal ischemia and reperfusion. Acta Cir Bras.

[7] Biederman J, Yee J, Cortes P (2004). Validation of internal control genes for gene expression analysis in diabetic glomerulosclerosis. Kidney Int.

[8] Billeschou A, Hunt JE, Ghimire A, Holst JJ, Kissow H (2021) Intestinal Adaptation upon Chemotherapy-Induced Intestinal Injury in Mice Depends on GLP-2 Receptor Activation. Biomedicines 9. 10.3390/biomedicines9010046 10.3390/biomedicines9010046 PMC7825593 33430185

[9] Cani PD, Bibiloni R, Knauf C, Waget A, Neyrinck AM, Delzenne NM, Burcelin R (2008). Changes in gut microbiota control metabolic endotoxemia-induced inflammation in high-fat diet-induced obesity and diabetes in mice. Diabetes.

[10] Cao Q, McIsaac SM, Stadnyk AW (2012). Human colonic epithelial cells detect and respond to C5a via apically expressed C5aR through the ERK pathway. Am J Physiol Cell Physiol.

[11] Coughlan MT, Sharma K (2016). Challenging the dogma of mitochondrial reactive oxygen species overproduction in diabetic kidney disease. Kidney Int.

[12] Ding J, Yu HL, Ma WW, Xi YD, Zhao X, Yuan LH, Feng JF, Xiao R (2013). Soy isoflavone attenuates brain mitochondrial oxidative stress induced by β-amyloid peptides 1–42 injection in lateral cerebral ventricle. J Neurosci Res.

[13] Elgendy D, Othman A, El-Guindy D, Soliman N, Elmehy D (2020) Synergy Between Beta-1, 3-Glucan and Praziquantel In The Treatment Of Experimental Chronic Schistosomiasis Mansoni. J Egypt Soc Parasitol 50:10. 10.21608/jesp.2020.131088

[14] Fang Q, Zhao X (2017). Clinical effect of combined ulinastatin and continuous renal replacement therapy on management of severe sepsis with acute kidney injury. Trop J Pharm Res.

[15] Guha M, Kumar S, Choubey V, Maity P, Bandyopadhyay U (2006). Apoptosis in liver during malaria: role of oxidative stress and implication of mitochondrial pathway. FASEB J.

[16] Ha H, Lee HB (2000). Reactive oxygen species as glucose signaling molecules in mesangial cells cultured under high glucose. Kidney Int Suppl.

[17] Horváth IL, Bunduc S, Fehérvári P, Váncsa S, Nagy R, Garmaa G, Kleiner D, Hegyi P, Erőss B, Csupor D (2022). The combination of ulinastatin and somatostatin reduces complication rates in acute pancreatitis: a systematic review and meta-analysis of randomized controlled trials. Sci Rep.

[18] Huang W, Man Y, Gao C, Zhou L, Gu J, Xu H, Wan Q, Long Y, Chai L, Xu Y, Xu Y (2020). Short-Chain Fatty Acids Ameliorate Diabetic Nephropathy via GPR43-Mediated Inhibition of Oxidative Stress and NF-kappaB Signaling. Oxid Med Cell Longev.

[19] Jha JC, Banal C, Chow BS, Cooper ME, Jandeleit-Dahm K (2016). Diabetes and Kidney Disease: Role of Oxidative Stress. Antioxid Redox Signal.

[20] Jiang L, Yang L, Zhang M, Fang X, Huang Z, Yang Z, Zhou T (2013). Beneficial effects of ulinastatin on gut barrier function in sepsis. Indian J Med Res.

[21] Joesten WC, Short AH, Kennedy MA (2019) Spatial variations in gut permeability are linked to type 1 diabetes development in non-obese diabetic mice. BMJ Open Diabetes Res Care 7:e000793. 10.1136/bmjdrc-2019-000793 10.1136/bmjdrc-2019-000793 PMC6936454 31908796

[22] Kitada M, Ogura Y, Koya D (2016). Rodent models of diabetic nephropathy: their utility and limitations. Int J Nephrol Renov Dis.

[23] Li L, Wei T, Liu S, Wang C, Zhao M, Feng Y, Ma L, Lu Y, Fu P, Liu J (2021). Complement C5 activation promotes type 2 diabetic kidney disease via activating STAT3 pathway and disrupting the gut-kidney axis. J Cell Mol Med.

[24] Lin D, Zhu X, Li J, Yao Y, Guo M, Xu H (2020). Ulinastatin alleviates mitochondrial damage and cell apoptosis induced by isoflurane in human neuroglioma H4 cells. Hum Exp Toxicol.

[25] Livak KJ, Schmittgen TD (2001). Analysis of relative gene expression data using real-time quantitative PCR and the 2(-Delta Delta C(T)) Method. Methods (San Diego, Calif).

[26] Lowry OH, Rosebrough NJ, Farr AL, Randall RJ (1951). Protein measurement with the Folin phenol reagent. J Biol Chem.

[27] Lu CC, Hu ZB, Wang R, Hong ZH, Lu J, Chen PP, Zhang JX, Li XQ, Yuan BY, Huang SJ, Ruan XZ, Liu BC, Ma KL (2020). Gut microbiota dysbiosis-induced activation of the intrarenal renin-angiotensin system is involved in kidney injuries in rat diabetic nephropathy. Acta Pharmacol Sin.

[28] Maity P, Bindu S, Dey S, Goyal M, Alam A, Pal C, Mitra K, Bandyopadhyay U (2009). Indomethacin, a non-steroidal anti-inflammatory drug, develops gastropathy by inducing reactive oxygen species-mediated mitochondrial pathology and associated apoptosis in gastric mucosa: a novel role of mitochondrial aconitase oxidation. J Biol Chem.

[29] Marrero MB, Banes-Berceli AK, Stern DM, Eaton DC (2006). Role of the JAK/STAT signaling pathway in diabetic nephropathy. Am J Physiol Renal Physiol.

[30] Matsumoto N, Riley S, Fraser D, Al-Assaf S, Ishimura E, Wolever T, Phillips GO, Phillips AO (2006). Butyrate modulates TGF-beta1 generation and function: potential renal benefit for Acacia(sen) SUPERGUM (gum arabic)?. Kidney Int.

[31] McClemens J, Kim JJ, Wang H, Mao YK, Collins M, Kunze W, Bienenstock J, Forsythe P, Khan WI (2013). Lactobacillus rhamnosus ingestion promotes innate host defense in an enteric parasitic infection. Clin Vaccine Immunol.

[32] Sharma P, Kaushik P, Jain S, Sharma BM, Awasthi R, Kulkarni GT, Sharma B (2021). Efficacy of Ulinastatin and Sulforaphane Alone or in Combination in Rat Model of Streptozotocin Diabetes Induced Vascular Dementia. Clin Psychopharmacol Neurosci.

[33] Shekarforoush S, Fatahi Z, Safari F (2016). The effects of pentobarbital, ketamine-pentobarbital and ketamine-xylazine anesthesia in a rat myocardial ischemic reperfusion injury model. Lab Anim.

[34] Shenouda SM, Widlansky ME, Chen K, Xu G, Holbrook M, Tabit CE, Hamburg NM, Frame AA, Caiano TL, Kluge MA, Duess MA, Levit A, Kim B, Hartman ML, Joseph L, Shirihai OS, Vita JA (2011). Altered mitochondrial dynamics contributes to endothelial dysfunction in diabetes mellitus. Circulation.

[35] Shikata K, Makino H (2013). Microinflammation in the pathogenesis of diabetic nephropathy. J Diabetes Investig.

[36] Souza CS, de Sousa Oliveira BS, Viana GN, Correia TML, de Braganca AC, Canale D, Oliveira MV, de Magalhaes ACM, Volpini RA, de Brito Amaral LS, de Jesus ST (2019). Preventive effect of exercise training on diabetic kidney disease in ovariectomized rats with type 1 diabetes. Exp Biol Med (Maywood).

[37] Sureshbabu A, Ryter SW, Choi ME (2015). Oxidative stress and autophagy: crucial modulators of kidney injury. Redox Biol.

[38] Tabit CE, Chung WB, Hamburg NM, Vita JA (2010). Endothelial dysfunction in diabetes mellitus: molecular mechanisms and clinical implications. Rev Endocr Metab Disord.

[39] Tan SM, Snelson M, Ostergaard JA, Coughlan MT (2022). The Complement Pathway: New Insights into Immunometabolic Signaling in Diabetic Kidney Disease. Antioxid Redox Signal.

[40] Wang H, Zhang D, Qian H, Nie J, Wei J (2022). Effects of Ulinastatin on Myocardial Ischemia-Reperfusion Injury, Cardiac Function, and Serum TNF-α and IL-10 Levels in Patients Undergoing Cardiac Valve Replacement under Cardiopulmonary Bypass. Comput Math Methods Med.

[41] Wang H, Liu B, Tang Y, Chang P, Yao L, Huang B, Lodato RF, Liu Z (2019). Improvement of Sepsis Prognosis by Ulinastatin: A Systematic Review and Meta-Analysis of Randomized Controlled Trials. Front Pharmacol.

[42] Wang P, Wang T, Zheng X, Cui W, Shang J, Zhao Z (2021). Gut microbiota, key to unlocking the door of diabetic kidney disease. Nephrology (Carlton).

[43] Wang WK, Lu QH, Wang X, Wang B, Wang J, Gong HP, Wang L, Li H, Du YM (2017). Ulinastatin attenuates diabetes-induced cardiac dysfunction by the inhibition of inflammation and apoptosis. Exp Ther Med.

[44] Yu T, Robotham JL, Yoon Y (2006). Increased production of reactive oxygen species in hyperglycemic conditions requires dynamic change of mitochondrial morphology. Proc Natl Acad Sci U S A.

[45] Zhang PN, Zhou MQ, Guo J, Zheng HJ, Tang J, Zhang C, Liu YN, Liu WJ, Wang YX (2021). Mitochondrial Dysfunction and Diabetic Nephropathy: Nontraditional Therapeutic Opportunities. J Diabetes Res.

[46] Zhao G, Zhu Y, Yu D, Ma J (2015). The effect of ulinastatin on hyperglycemia in patients undergoing hepatectomy. J Surg Res.

